# Genomic characterization of the conditionally dispensable chromosome in *Alternaria arborescens* provides evidence for horizontal gene transfer

**DOI:** 10.1186/1471-2164-13-171

**Published:** 2012-05-06

**Authors:** Jinnan Hu, Chenxi Chen, Tobin Peever, Ha Dang, Christopher Lawrence, Thomas Mitchell

**Affiliations:** 1 Department of Plant Pathology, The Ohio State University, Columbus, OH 43210, USA; 2 Department of Plant Pathology, Washington State University, Pullman, WA 99164-6430, USA; 3 Virginia Bioinformatics Institute, Virginia Polytechnic Institute and State University, Blacksburg, VA 24061, USA

**Keywords:** *Alternaria arborescens*, Illumina sequencing, Conditionally dispensable chromosome, Horizontal gene transfer, Polyketide synthase, Host specific toxins

## Abstract

**Background:**

Fungal plant pathogens cause serious agricultural losses worldwide. *Alternaria arborescens* is a major pathogen of tomato, with its virulence determined by the presence of a conditionally dispensable chromosome (CDC) carrying host-specific toxin genes. Genes encoding these toxins are well-studied, however the genomic content and organization of the CDC is not known.

**Results:**

To gain a richer understanding of the molecular determinants of virulence and the evolution of pathogenicity, we performed whole genome sequencing of *A. arborescens*. Here we present the *de-novo* assembly of the CDC and its predicted gene content. Also presented is hybridization data validating the CDC assembly. Predicted genes were functionally annotated through BLAST. Gene ontology terms were assigned, and conserved domains were identified. Differences in nucleotide usage were found between CDC genes and those on the essential chromosome (EC), including GC3-content, codon usage bias, and repeat region load. Genes carrying PKS and NRPS domains were identified in clusters on the CDC and evidence supporting the origin of the CDC through horizontal transfer from an unrelated fungus was found.

**Conclusions:**

We provide evidence supporting the hypothesis that the CDC in *A. arborescens* was acquired through horizontal transfer, likely from an unrelated fungus. We also identified several predicted CDC genes under positive selection that may serve as candidate virulence factors.

## Background

The rapid development of next-generation sequencing technologies over the past decade has led to a flood of both *de-novo* sequencing and re-sequencing projects in almost every branch of the tree of life. Within the fungal kingdom, comparative genome studies have led to the unexpected finding that large genomic regions may be variable among isolates of a given species. One category of these variable regions are unique chromosomes referred to as supernumerary or conditionally dispensable because they are not typically required for saprophytic growth [1-3]. These chromosomes have been identified in many fungi including *Magnaporthe oryzae *[4-6], *Fusarium oxysporum *[7], *Nectria haematococca *[8,9], *Mycosphaerella graminicola *[10], *Cochliobolus heterostrophus *[11], *Leptosphaeria maculans *[12], and *Alternaria alternata *[13,14].

Plant pathogenic fungi in the genus *Alternaria * infect a remarkable range of host plants and are major causes of agricultural yield losses [15]. Conditionally dispensable chromosomes (CDCs) are carried by several of the small-spored, plant-pathogenic *Alternaria * species [13,14,16]. These chromosomes are generally less than 2.0MB in size, and may be transmitted horizontally between isolates in a population, potentially conferring new pathogenic attributes to the receiving isolate [17-20]. Loss of the CDC can also occur during repeated sub-culturing, resulting in the transition from a pathogenic to saprophytic form of the fungus [13]. Several genes coding host specific toxins (HSTs) have been located to gene clusters on CDCs, including those producing AF-toxin from the strawberry pathotype [21], AK-toxin from the Japanese pear pathotype [22], and ACT-toxin from the tangerine pathotype [23]. These toxins share a common 9,10-epoxy-8-hydroxy-9-methyl-decatrienoic acid structural moiety, with the genes encoding each toxin sharing a high degree of homology [21-25]. In addition, the AMT gene from the apple pathotype, a gene involved in host-specific AM-toxin cyclic peptide biosynthesis, is located on a small chromosome of 1.1 to 1.7 Mb [13,26], with at least four copies involved in AM-toxin biosynthesis [27]. The only other gene sequences identified to date on CDCs are extended families of transposon-like sequences (TLSs) [14].

Horizontal gene transfer (HGT) is the movement, without recombination, of stable genetic material between two individuals [28]. HGT may not only occur between different individuals of the same species, but also between species or even between bacteria and fungi or between fungi and oomycetes [29,30]. In fungi, the movement of plasmids, mycoviruses, transposable elements, gene clusters, and whole chromosomes have been demonstrated from one individual to another [31]. The first theory to explain gain and loss of HSTs was proposed in 1983 [32]. It has then been hypothesized that the genome content of CDCs in *Alternaria* species were acquired through HGT events [14]. The most well studied example of HGT in fungi is the movement of the *ToxA* gene from the wheat blotch pathogen *Stagonospora nodorum* to *Pyrenophora tritici-repentis*, the causal agent of tan spot of wheat [33,34]. This horizontal transfer event was identified by nucleotide sequence similarity and structural comparisons between genes from both species. The direction of transfer was inferred by the fact that the *ToxA* gene consisted of a single haplotype in *P. tritici-repentis* but 11 haplotypes in *S. nodorum* isolates.

*Alternaria arborescens* (synonym *A. alternata* f. sp. *lycopersici*), the fungus that produces host-specific AAL toxin, is the causal agent of stem canker of tomato [35,36]. It has been observed in pulsed field gel electrophoresis (PFGE) studies that *A. arborescens* carries one CDC of 1.0-Mb [16,37]. To date, only two genes have been reported to be carried on this CDC including *ALT1*, which is a PKS gene involved in AAL toxin biosynthesis [38,39], and *AaMSAS*, also a PKS gene [40,41]. A CDC deletion mutant of *A. arborescens* generated through restriction enzyme mediated integration (REMI) showed a toxin and pathogenicity minus phenotype [41]. In addition, in protoplast fusion experiments, a CDC from *A. arborescens* was observed to transfer into the strawberry pathotype, and subsequently introduced new tomato pathogenicity to the fusant [41].

In this study, we used a next generation sequencing approach to produce a draft sequence of the *A. arborescens* genome and used a novel bioinformatics approach to separate CDC contigs from the essential chromosome (EC) contigs. The gene content of the CDC was analyzed to answer the following questions: (1) What is the difference between the CDC and EC genome content at the nucleotide level? (2) Are CDC genes under positive selection and could they represent additional virulence factors in addition to the known toxin encoding genes? (3) Is the evolutionary history of the CDC the same as that of the ECs, and is there any evidence of a HGT event? In answering these questions, we confirmed a different genome content pattern of the *A. arborescens* CDC and found evidence for HGT.

## Results

### Sequencing & assembly

*A. arborescens* strain EGS 39–128 (CBS 102605) [42] was sequenced by a whole genome shotgun approach using the Illumina Genome Analyzer II, which resulted in ~50 million paired-end short reads of 75 bp representing 90X average coverage of the predicted genome content. *De-novo* assembly was performed using Velvet [43] (version 0.7), and confirmed by Edena [44] and Minimus2 [45]. The assembly resulted in 1,332 contigs with a N50 of 624KB and total size of 34.0MB ( Additional file 1: Table S1; Assembly has been deposited at DDBJ/EMBL/GenBank under the accession AIIC00000000. The version described in this paper is the first version, AIIC01000000.) One hundred thirty-seven large contigs with lengths greater than 10KB and representing 98% of the genome assembly content were chosen for further analysis.

### Marker-assisted identification of contigs carrying toxin biosynthetic genes

**Figure 1:**
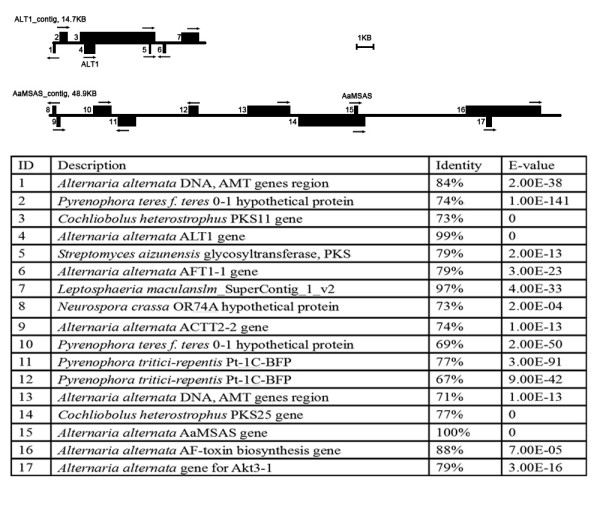
**Annotation of the initial two CDC contigs.** The two lines represent two contigs containing CDC marker genes. Each box represents a BLAST annotation with the direction indicated by arrows.

The first challenge in analyzing the CDC was to isolate its assembly contigs away from EC contigs. For this genome, the process was made more challenging as there is no defined reference genome, few genetic markers, and no optical map. It is known from previous studies that most *Alternaria* species, including the isolate used in this study, have a single CDC [16,37]. To begin assembly of the this chromosome, two previously identified CDC genes that belong to the toxin biosynthetic cluster, *ALT1 * and *AaMSAS *[38,40], were used as markers to search in all contigs. Through this strategy, two putative CDC contigs of 15 KB and 48 KB in length were identified as containing *ALT1* and *AaMSAS*, respectively. These two contigs were annotated to identify PKS genes, other toxin biosynthetic genes, as well as genes with orthologs in other fungi and bacteria (Figure 1). Multiple putative HST genes were identified on both contigs, consistent with predictions based on previous reports [14].

### Identification of the remaining CDC contigs and validation by Southern hybridization

**Figure 2:**
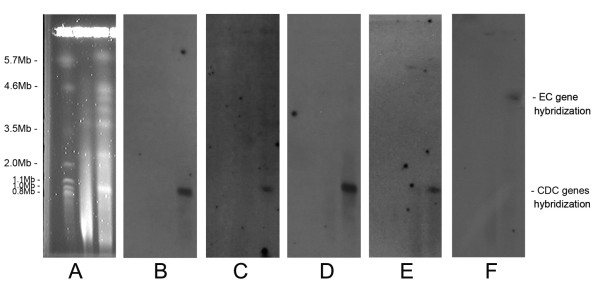
**Southern hybridization to validate CDC contigs prediction. ** (A) Chromosomes of *A. arborescens* separated by CHEF. Hybridization of (B) *ALT1* gene, three predicted CDC genes including (C) CDC_92, (D) CDC_102, (E) CDC_147, and (F) a predicted EC gene EC_97_90_g721 are shown. It should be noted that the membrane used for hybridization was prepared in 2004, so only a limited number of hybridization experiments were possible.

To identify additional CDC contigs, the *Alternaria brassicicola* ( *Ab*) genome sequence was used as a reference (downloaded from The Genome Institute at Washington University). *A. brassicicola* is a related species to *A. arborescens* ( *Aa*) but does not carry CDCs. All contigs from *Aa* were aligned to *Ab* contigs using MUMmer [46] as the alignment tool with an identity cut-off at 90%. Eight previously identified marker genes from *Aa*, 6 from the EC and 2 from CDC, were used to set criteria to distinguish contigs belonging to CDCs versus ECs [41]. (Table 1) After comparing the alignments of contigs containing the 8 marker genes, we set a CDC contig cut-off as those contigs with less than 20% coverage of sequence aligned to *Ab* with higher than 90% identity (including both coding and non-coding regions). Through this method, 29 predicted CDC contigs were identified with total length of 1.0Mb, the same as the expected size from clamped homogenous electric fields (CHEF) gel analysis [16,37] (Figure 2A). The remaining 108 contigs were considered essential chromosomes (ECs) with total genome size of 32.3MB ( Additional file 2: Figure S1). Validation that the selected contigs belong to the CDC was performed by Southern hybridization using genes predicted to reside on the CDC and EC contigs respectively as probes (Figure 2C-F). Five probes were hybridized including 4 from the CDC and 1 from the EC. Each probe gene is predicted to be present in a single copy. The first probe was *ALT1*, a toxin gene known to reside on the CDC [41]. This was followed by hybridizing with three CDC genes predicted from the *de-novo* assembly, including a transporter CDC_92, a polysaccharide export protein CDC_102, and an o-methyltransferase CDC_147. The fifth probe was EC_97_90_g721, a gene annotated as PKS and predicted to reside on the ECs.

**Table 1:** Alignment of *A. arborescens* marker gene contigs and *A. brassicicola* contigs

**Contig ID**	**Length (bp)**	**Alignment coverage**	**Marker gene**	**New ID**	**GC%**
NODE_9	48862	13.6%	*AaMSAS*	CDC_contig_23	53.015%
NODE_136	14729	15.7%	*ALT1*	CDC_contig_27	24.942%
NODE_758	174048	26.1%	*AKS17*	EC_contig_009	51.127%
NODE_8	199480	44.1%	*AKS21*	EC_contig_038	51.143%
NODE_58	833831	61.9%	*MAT1-2-1*	EC_contig_063	51.209%
NODE_82	1201916	69.9%	*ALM*	EC_contig_085	51.495%
NODE_274	1472031	72.1%	*VKS2*	EC_contig_091	51.489%
NODE_151	812677	73.5%	*AaTUB*	EC_contig_094	51.705%

The first two contigs contain two CDC marker genes and the following six contigs contain six EC marker genes.

### Gene prediction, length, GC3-content, and repeat identification

Nine thousand, one hundred sixty-seven genes were predicted by FGENESH [47] using pre-trained *Alternaria* parameters, of which 209 genes were assigned to CDC contigs and 8958 to EC contigs. The average length of each predicted gene was 1.8 KB, and the gene density was 3.7KB per gene. Compared to gene predictions for *A. brassicicola* (average gene length = 1.3KB, gene density = 3.0KB per gene), *A. arborescens* genes were longer and present in lower density. To evaluate the origin of the CDC, the predicted genes residing on the CDC and EC contigs were compared at the nucleotide level, including gene length, GC3-content [48], repeat load, and codon usage bias. This analysis showed that CDC genes are about 200bp shorter on average than EC genes (*P* = 2.36E-09) and have significantly lower GC3-content ( *P* = 0.028). Repeat regions composed 5.3% of CDC contigs while only 0.6% of EC contigs (Additional file 1: Table S2). It should be noted that some repeat regions could be lost in short read sequences *de-novo* assemblies, however, even with possible suppressed numbers, this result indicates approximately 10X repeat enrichment in the CDC compared to the EC.

### Codon usage analysis

**Figure 3:**
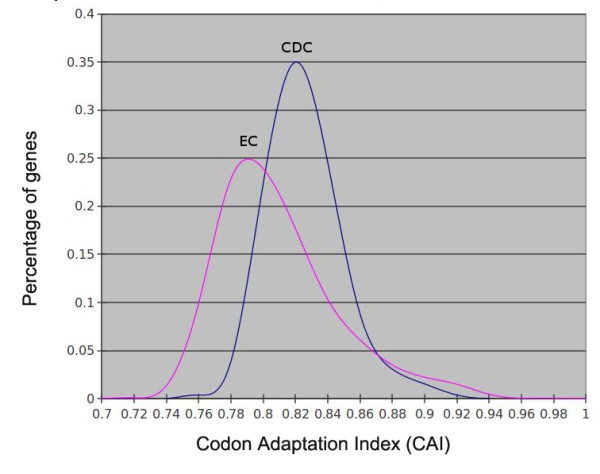
**The Codon Adaptation Index (CAI) distribution of*A. arborescens *CDC genes compared to EC genes.** The CAI derived from the RSCU estimations was computed using “Automated Codon Usage Analysis Software” (ACUA).

Codon usage comparisons of CDC and EC genes were used to determine whether a bias in codon usage exists between the two groups. Both the Codon Adaptation Index [49] (CAI, *P* = 0, Figure 3) and Relative Synonymous Codon Usage (RSCU) [50] correlation (*P* = 1.14E-14, Additional file 1: Table S3) from the two groups were significantly different, suggesting a different origin. The largest codon usage bias was observed for the amino acids Tyrosine, Lysine, and Asparagine, with a preference for A over G and T over C in CDC genes (Additional file 1: Table S4). These three amino acids were not biased for CDCs in *Fusarium*[7], indicating there's no universal CDC codon usage bias pattern between *Alternaria* and  *Fusarium*.

### Annotation of EC genes

The assembly results showed the size of essential chromosomes region collectively to be 33.0 MB with 8958 predicted genes. RepeatMasker identied only 0.12% of the EC region as simple repeats (about 50bp in length) and 0.08% as low complexity, indicating that short repeats may be lost during *de novo* assembly of Illumina sequencing reads. For secreted protein identification, 1099 (12.2%) of the EC proteins were predicted to contain signal peptides, and were functionally annotated using BLAST to the NCBI database with more than 98% of the genes returning at least one hit with an E-value < 1.0E-3. From the BLAST results, we identified 212 transcription factors, 98 oxidase proteins, 202 kinase proteins, 279 transporters, 81 Cytochrome P450s, and 45 different proteases.

### Annotation of CDC genes

Several host-specific toxin genes and transposon-like sequences have been reported to be carried by CDCs in *Alternaria *[14]. We used two methods to annotate the functions of resident CDC genes: (1) they were blasted against the NCBI non-redundant database as well as Pfam [51] and NCBI CDD [52] to search for functional domains; (2) they were scanned to identify transcription factors, PKS genes, NRPS genes, P450s, transporters, and pathogenicity related genes.

**Figure 4:**
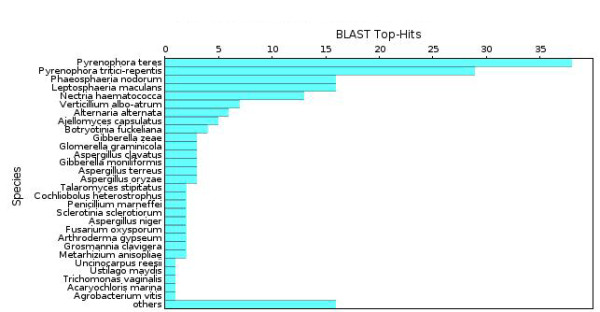
**BLAST taxonomy report of all CDC genes against NCBI “Nucleotide collection (nr/nt)” database. ** Species of BLAST hits (E-value < 1.0E-3) were ranked by their occurrence.

From 160 NCBI BLASTN hits of putative CDC genes (Figure 4), the top five species matches were *Pyrenophora teres*, *Pyrenophora tritici-repentis*, *Phaeosphaeria nodorum*, *Leptosphaeria maculans*, and *Nectria haematococca*, all of which are fungal phytopathogens. Interestingly, *A. alternata* was ranked 7^th^ in this list, demonstrating that CDC genes were more similar to genes present in other fungal species rather than other *Alternaria* spp. Moreover, besides *N. haematococca*, all these other fungi are closely related taxonomically belonging to the class, Dothideomycetes.

Gene ontology terms were assigned to CDC genes based on BLAST matches with sequences whose function was previously characterized [53]. Ninety CDC genes were assigned to a biological process, 51 for molecular function, and 15 for cellular component (Additional file 2: Figure S2). Among the biological process assignments, 54% of genes were assigned to “metabolic process”, and 10% to “biosynthetic process”. Enrichment of metabolic and biosynthetic process in CDC genes as compared to EC genes supported the observation that *Alternaria* CDC genes were enriched for polyketide synthases (PKS) and toxin synthases. Molecular function terms showed a significant percentage (39%) to “nucleotide/nucleic acid binding”, which showed an enrichment of transcription factors and gene regulation elements.

To provide a more detailed characterization of putative CDC genes, each was translated to identify protein families. Among the 209 predicted CDC proteins, 31 were identified as carrying PKS domains. Two proteins were found to carry highly modular domains: KS-AT-KR-ACP on CDC_141 and KS-AT-DH-ER-KR-ACP on CDC_165. The remaining 29 PKS proteins each carried 1 or 2 ACPs (Acyl carrier protein) domains. Seven proteins were found to carry NRPS domains: 3 Enterobactin domains, 2 Bacitrancin domains, 1 Pyochelin domain, and 1 CDA1 domain. Two proteins were identified as hybrid PKS-NRPS. Seven proteins were identified as P450 monooxygenase proteins. For transcription factors, 24 proteins were characterized to contain TF domains, in which Zn2Cys6 was the prominent group. Multiple ADP/ATP transporters, ABC transporters, ion transporters and major facilitator superfamily (MFS) transporters were also found in CDC protein group. Additionally, it was found that multiple proteins carrying FAD binding domains and oxidoreductases. Finally, 37 proteins were identified as putative pathogenicity related genes through scanning CDC genes in the pathogen-host interactions database (PHI-base) [54] (E-value < 0.05). See Additional file 3 for a complete CDC gene annotation list.

### Secondary metabolite biosynthetic gene clusters

**Figure 5:**
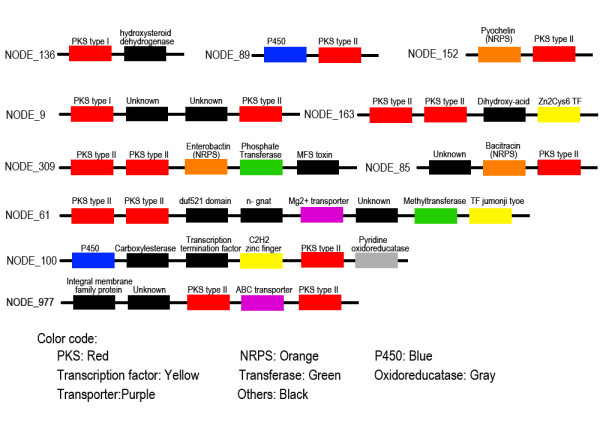
**Structure of predicted secondary metabolite biosynthesis (SMB) clusters. **Each block represents a predicted CDC gene with the annotation listed above the block. Contig (NODE) numbers and color legend are given. Type I PKSs are multifunctional enzymes that are organized into modules, each harbors a set of distinct, non-iteratively acting activities responsible for the catalysis, while type II PKSs are multi-enzyme complexes carrying a single set of iteratively acting activities.

In fungi, it has been reported that genes responsible for secondary metabolite biosynthesis (SMB) may be clustered [4,55]. Typically these include PKS or NRPS genes, as well as genes responsible for structural modifications of initial metabolites, for transport, and for transcription regulation [56]. In this study, we screened each CDC gene and those surrounding them, looking for evidence of clustering of PKS, NRPS, transcription factors, transporters, P450 proteins, FAD binding proteins, transferases, and oxidoreductases. We identified 10 putative SMB clusters (Figure 5). A typical SMB cluster is formed by 3–6 genes, with 1 or 2 PKS or NRPS genes, and other metabolite syntheses related genes.

### Evolutionary selection of CDC genes and domains

**Figure 6:**
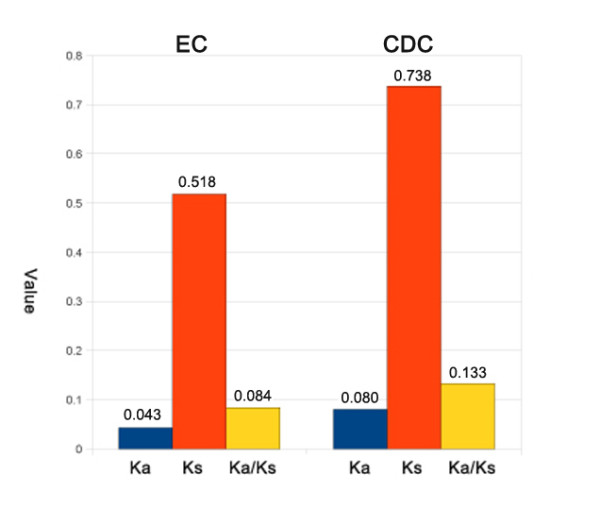
**Average Ka/Ks ratio, Ka, and Ks for all aligned EC genes and CDC genes. **6323 EC proteins and 34 CDC proteins were involved and Codem program in the PAML suite was used (Capital “C”, add “was”).

To estimate selection on CDC and EC genes, Ka/Ks ratios were calculated, with the assumption that genes with Ka > Ks were likely under positive selection, genes with Ka = Ks were likely evolving neutrally, and genes with Ka < Ks were likely under purifying (negative) selection. Twenty-eight CDC and 6,036 EC genes were successfully aligned to *A. brassicicola* genes and Ka and Ks values were calculated for each. It was observed CDC genes had about a double Ka (0.08/0.043) and larger Ka/Ks ratios (0.133/0.084) than EC genes (Figure 6), possibly indicating greater positive selection on CDC genes. The two CDC genes with highest Ka/Ks ratio was CDC_102 (PKSs) and CDC_146 (phosphotransferase). However, no CDC genes showed Ka/Ks > 1, suggesting that in these two *Alternaria* species strong positive selection may only occur in specific regions of a protein. The selection ratio only at conserved domains of CDC genes was then estimated. Domains of aligned CDC genes were identified using the NCBI CDD database. Each individual domain was extracted then the Ka/Ks ratio was calculated and compared to that from same full length protein (Additional file 1: Table S5). We found two domains from CDC_151 with a higher Ka/Ks ratio compared to whole length protein: a 12x increase for the haloacid dehalogenase-like hydrolases (HAD_like) domain, which uses a nucleophilic aspartate in their phosphoryl transfer reaction, and 2x increase for heavy-metal-associated (HMA) domain, which transports or detoxifies heavy metals. Another interesting example was CDC_144, whose domain patatin-like phospholipase (Pat17, belonging to the alpha-beta hydrolase family) showed a 1.5x increase compared to whole length protein.

### Origin of CDC

**Figure 7:**
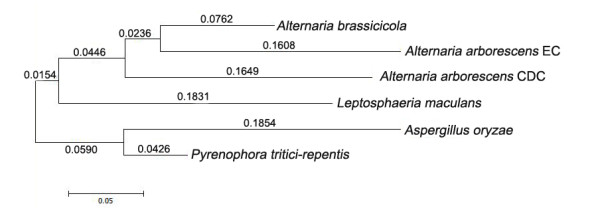
**Phylogenetic relationship of proteins in*A. arborescens*CDC shows discordance compared to EC proteins. **The phylogenetic distance tree was constructed by “MEGA 5” using six protein sequences showing homology in all six groups.

In the taxonomy report of CDC BLAST results, top hits came primarily from closely related dothideomycete fungi, indicating CDC may have fungal origin, or a transfer event occurred between CDC content and one or more fungal genomes. To test whether CDCs have the same phylogenetic placement with ECs, a phylogenetic analysis was conducted, including EC and CDC genes from *A. arborescens*, genes from *A. brassicicola*, and from three other ascomycete species: *P. tritici-repentisL. maculans*, and *A. oryzae*. Proteins coded by 6 genes showing homology in all 6 groups were used to build a distance tree (Figure 7) using the neighbor-joining method [57]. Results show the CDC clade was within but basal to the two *Alternaria* clades.

**Figure 8:**
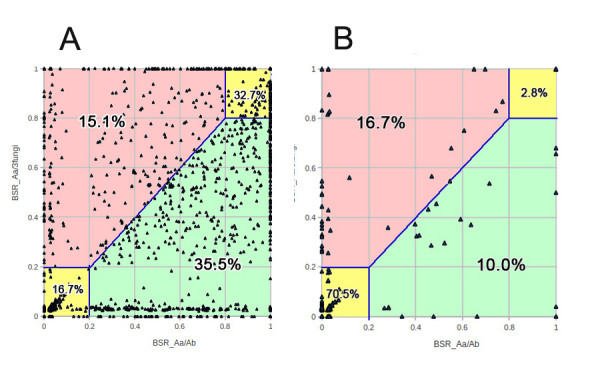
**Scatter plots of BLAST Score Ratio (BSR) of EC proteins (A) and CDC proteins (B). **The numbers in yellow regions indicate the percentage of genes that either lack homologous sequences (lower left corner) or contain homologous sequences in both *A. brassicicola* ( *Ab*) library and 3-fungi library (upper right corner). The numbers in pink regions (upper left) indicate the percentage of genes with homologous sequences in 3-fungi library but not in *Ab* library, while numbers in green regions (lower right) indicate the percentage of genes with homologous sequences in the *Ab* library but not in the 3-fungi library.

A BLAST score ratio (BSR) [58] analysis was performed to test whether individual proteins on the *A. arborescens* CDC had more similarity to *A. brassicicola* or other fungi, and the result was compared with the same analysis to EC proteins. Complete genome protein sequences of three fungal species: *P. tritici-repentisL. maculans*, and *A. oryzae* were extracted and built into a library called “3-fungi”, representing proteins from closely related fungal species. Then proteins from the CDC and ECs were compared to the *A. brassicicola* protein library and “3-fungi” protein library respectively (Figure 8). It was clear, that there was less divergence between EC proteins and *A. brassicicola* proteins compared to that with other fungi (35.5% vs 15.1%), consistent with the species phylogeny. In contrast, CDC proteins were more similar to proteins from other fungi (16.7% vs 10.0%), suggesting they have different evolutionary history other than EC proteins.

## Discussion

### Comparison to other CDC containing fungi

Compared to other recently published assemblies of CDCs in filamentous fungi, *A. arborescens* has a relatively small number of CDCs (one) and the size (1.0Mb) is small. *M. graminicola * was reported to have the highest number of dispensable chromosomes with upwards of 8 ranging in size from 0.39 to 0.77MB [59]. Three CDCs in *N. haematococca *[9,60], and 4 complete CDCs and partial region of another 2 in *F. oxysporum*[7] were identified. In other *Alternaria * species, identified CDCs are relatively larger such as 1.05Mb in the strawberry pathotype [14], 1.1 to 1.7 Mb (depending on strains) in the apple pathotype [13], and 4.1 Mb in the Japanese pear pathotype [61,62]. In *A. arborescens*, only 1 dispensable chromosome is present, representing only 3% of the genome content, which is significantly smaller than other cases and may suggest a more recent acquisition or different origin.

### PKS and NRPS clusters

Phytopathogenic fungi produce a diverse array of secondary metabolites, including host-selective toxins conferring pathogenicity [63]. It was reported in two basidiomycete maize pathogens candidate effector genes were located in small clusters that were dispersed throughout (both "are" change to "were") the genome [64]. However, in some other fungi, especially ascomycetes, genes coding for toxins can co-locate in clusters consisting of more than 10 contiguous genes. A well-known example is the trichothecene biosynthetic gene cluster in *F. graminearum* which contains 10–12 genes including a terpene synthase gene, P450 monooxygenase genes, acyl transferase genes, regulatory genes, and transporter genes [55]. While in *A. fumigatus,* 26 SMB clusters were identified, each containing 5–48 genes [65]. In our study, 29 PKS, 5 NRPS, and 2 hybrid PKS-NRPS genes were found on the CDC, with larger density compared to other fungi. However, among 10 predicted SMB clusters, most were relatively small and only carried 3–8 genes, which may not represent the true cluster size due to short contigs length that may divide one large cluster into two or more. One example of an identified cluster was located on contig Node_309 which consists of 5 genes, including 2 PKSs, 1 NRPS putatively coding for enterobactin, a phosphate transferase gene, and a MFS transporter. It lacks regulators, P450s, and transporters compared to other typical clusters. However, this cluster locates at the edge of the contig. Only 5 genes away from this cluster, another small cluster containing PKS, NRPS, P450s and an ABC transporter was identified, suggesting these two could be part of a larger cluster (see Additional file 3: Supplementary CDC annotation list). In this study, PKS genes were identified by screening the PKS sequence database, especially the domain database, which include: ketoacyl synthase (KS), acyl transferase (AT), ketoreductase (KR), dehydratase (DH), enoyl reductase (ER), and acyl carrier protein (ACP, also known as PP domain). The KS, AT, and ACP domains are essential for PKS genes [66]. Two PKS genes were identified to have multiple domains above: KS-AT-KR-ACP in CDC_141, and KS-AT-DH-ER-KR-ACP in CDC_165. The remaining 29 PKS genes each carries 1 or 2 ACP domains. Despite these conserved domains, other domains carried by these genes were divergent, indicating variance and multifunction of each PKS genes (Additional file 1: Table S6). However, at least 3 domain families were found to be enriched in the indentified PKS genes: ABC_membrane (4 identified), NADB_Rossmann (7 identified), and P-loop NTPase (6 identified), suggesting these proteins are transmembrane and catalyzing enzymatic reactions. In the NRPS and hybrid PKS-NRPS gene group, enterobactin, bacitracin, pyoverdine, syringomycin, and CDA1 domains were identified, 4 of which were reported from to bacteria [67-70]. We eliminated the possibility of these genes originating from bacterial sequencing contamination by BLAST comparing all assembly contig sequences against the NCBI All Bacterial database with 2017 genome sequences, and found that the species with most hits was *Streptomyces coelicolor* with > 80% identity. However, only 0.7% of the entire *S. coelicolor* genome was covered. Indicating that either these genes have an origin from bacteria or their product proteins interact with each other and require a highly conserved structure that was retained during evolution.

### Horizontal gene transfer

According to the horizontal gene transfer hypothesis, *A. arborescens* may have acquired its CDC from another *Alternaria* species, from a fungus other than *Alternaria*, or possibly from a bacterium or virus [71]. There are at least two other possible explanations for its origin: (1) CDCs were present in an *Alternaria* ancestor, but were independently lost during vertical transmission in other non-pathogenic *Alternaria* species. (2) CDCs arose from essential chromosome as a copy first but then went under divergence so no obvious orthology could be detected. To test which of the three models fits this case best, we built a complete EC protein library and blasted all CDC proteins against it to detect any possible orthology. Out of 209 CDC proteins, we found 12 (5.7%) showing orthology to EC proteins. Although the low orthology percentage alone could not exclude the “duplication and divergence” model, taken together with differences on GC3-content and codon usage bias, the possibility that this model fits is minimal.

To distinguish between HGT and vertical transmission hypothesis, we identified differences between *A. arborescens* CDC and EC genes in length, GC3-content, and codon usage bias. There was limited orthology detected between two groups; CDC genes showed discordant phylogenetic relation with EC, and had higher similarity to other fungi than *A. brassicicola*. From previous phylogenetic analysis of 13 *A. alternata* isolates collected worldwide, CDC genes from different isolates were almost identical despite diverse EC background [41]. Taken these results together, we concluded that the HGT model may serve as the best fit model in this case. Additionally, these data support the theory proposed in 1983 by Nishimura that Alternaria species acquired HSTs by HGT [32].

In this study, we identified evidence for the possibility of HGT event occurred in *A. arborescens*. For *Alternaria*, this strategy has its advantages. First, as a pathogen with a wide host range, as observed in nature, transportable pathogenicity chromosome may increase pathogen's adaptation to environment. Second, loss of a CDC when there's no host may reduce the cost of carrying extra genome content. Third, as asexual fungi, horizontal transfer may compensate the lack of genetic recombination.

## Conclusions

In this study, we identified *A. arborescens* CDC sequences through a whole genome sequencing and *de-novo* assembly process. By comparing nucleotide usage between CDC and EC contigs, we found evidence supporting HGT in *A. arborescens*. We also identified some predicted CDC genes under positive selection that may serve as virulence factors. However, questions still remain, such as the similarity and difference among CDCs from different *A. arborescens* isolates. To better understand CDC characteristics and mechanisms of HGT, other *Alternaria* isolates need to be sequenced.

## Materials and methods

### Sequencing, assembly & alignment

*A. arborescens* DNA was extracted following a protocol described [72] and the sequencing library was prepared using the Illumina Paired-End DNA Sample Prep Kit. Sequencing was performed using Illumina Genome Analyzer II. Short reads were assembled *de-novo* using Velvet, and assembly quality was improved by a pipeline including two alternate assemblers: Edena [44], and Minimus2 [45]. Parameters including k-mer length for Velvet and hash length for Edena were optimized by sequential step changes. The alignment between *A.arborescens* and *A.brassicicola* was conducted using the Nucmer program in the MUMmer suite [46], with parameter c = 15, l = 10. Alignments with identities lower than 90% or lengths shorter than 100bp were removed.

### Southern hybridization

On the CHEF gel membrane presented in Figure 2, lane 1 contains size markers, lane 2 contains *A. arborescens* chromosomes that had degraded, and lane 3 contains intact *A. arborescens* chromosomes. Southern hybridization was conducted using the GE health CDP-Star kit with 5 gene probes, including 1 CDC marker gene *ALT1*, 3 predicted CDC genes, and 1 predicted EC gene. Primers (Additional file 1: Table S7) were designed using Primer3 [73] (v0.4.0). Blots were stripped between hybridizations to ensure no probes from previous hybridization remained. Film was exposed for 48 hours.

### Gene prediction, codon usage analysis & repetitive DNA identification

Gene prediction was conducted using FGENESH [47], an *ab initio* gene predictor provided in the Softberry website. A pre-trained *Alternaria* matrix was used to optimize predictions. Both CDSs and protein sequences were generated and converted into fasta format files. ACUA [74] was used for calculating CAI and RSUC for each gene, and CAI distribution curves from the CDC group and EC group were compared to each other. Student’s F-test was used to test statistical significance. RepeatScout [75] was used for *de-novo* identification of repeat sequences in both CDC and EC sequences. The repeat libraries were then aligned back to CDC and EC contigs using Nucmer to calculate the repeat percentage for each group.

### Gene annotation

Blast2go [76] was used to annotate genes by “BLASTX” to the NCBI non-redundant protein database and then GO term assignment from the gene ontology database. Annotation of conserved domains was identified by scanning proteins through Pfam and NCBI CDD. PKS and NRPS genes were identified through scanning an online database SBSPKS [77]. The Fungal transcription factor database (FTFD) [78] was used to identify transcription factors. Transporters, P450s, and oxidoreducatases were identified based on BLAST and domain inspection. Potential secreted proteins were predicted using Signal 3.0 [79]. Pathogenicity and virulence factors were identified through scanning CDC genes in the pathogen-host interactions database (PHI-base) [54].

### Estimating Ka/Ks Ratios

*A. arborescens* proteins were blasted against *A. brassicicola* proteins to generate a match list between the two groups with a bits score cut-off at 300. The gene sequences coding for aligned proteins were extracted by an in-house PERL script. Prank [80] was used to conduct codon alignment, in which two protein sequences were aligned first and then DNA sequences were aligned based on the corresponding protein alignments. The codon alignment result was then entered into “Codem” in PAML [81] (v4.0) for Ka and Ks calculation with model M0. In calculating, the Nei and Gojobori [82] method and Yang and Nielsen [83] method were used.

## Abbreviations

CDC, Conditionally dispensable chromosome; EC, Essential chromosome; PKS, Polyketide synthase; NRPS, Nonribosomal peptide synthetase; HST, Host specific toxin; HGT, Horizontal gene transfer; PFGE, Pulsed field gel electrophoresis; REMI, Restriction enzyme mediated integration; CHEF, Clamped homogenous electric fields; CAI, Codon adaptation index; RSCU, Relative synonymous codon usage; CDD, Conserved domains database; SMB, Secondary metabolite biosynthesis; BSR, Blast score ratio.

## Competing interests

The authors declare that they have no competing interests.

## Authors’ contributions

JH, genome assembly, annotation, analysis, and writing; CC, Southern hybridization and writing; TP, fungal isolate, CHEF gel membrane, and editing; HD, CL, assisted with assembly validation and editing; TM, conceived and designed study, analysis, and writing. All authors read and approved the final manuscript.

## Supplementary Material

Additional file 1 ** Supplementary Tables contains: Table S1 to S7.** Table S1: Velvet *de-novo* assembly statistics. Table S2: Repeat region identification. Table S3: Codon usage correlation analysis. Table S4: Differences in codon usage between CDC and EC genes. Table S5: Ka/Ks ratio of CDC protein conversed domains. Table S6: Conserved domains in CDC putative PKS genes. Table S7: Primers used for Southern hybridization. Click here for file

Additional file 2 ** Supplementary Figures contains: Figure S1 to S2.** Figure S1: Global contig alignment between *A. arborescens* contigs and *A. brassicicola* contigs. Figure S2: GO term of CDC genes. Click here for file

Additional file 3 CDC gene annotation list: a spreadsheet with all annotation information for all CDC genes. Click here for file
