# Supplementary material for: Genomic characterization of the conditionally dispensable chromosome in Alternaria arborescens provides evidence for horizontal gene transfer
Source: BMC Genomics. 2012 May 6;13:171. doi: 10.1186/1471-2164-13-171 (PMC3443068; doi:10.1186/1471-2164-13-171)

Figure S1. Global contig alignment between *A. arborescens* contigs (blue blocks) and *A. brassicicola* contigs (red blocks). The first 11 lines contain EC contigs alignment and the last line (and the magnified inset) contains CDC contigs alignment. Alignment identity cut-off was set as 70%. Contig numbers are given above blocks. The corresponding alignment identity is shown as spots under contigs.

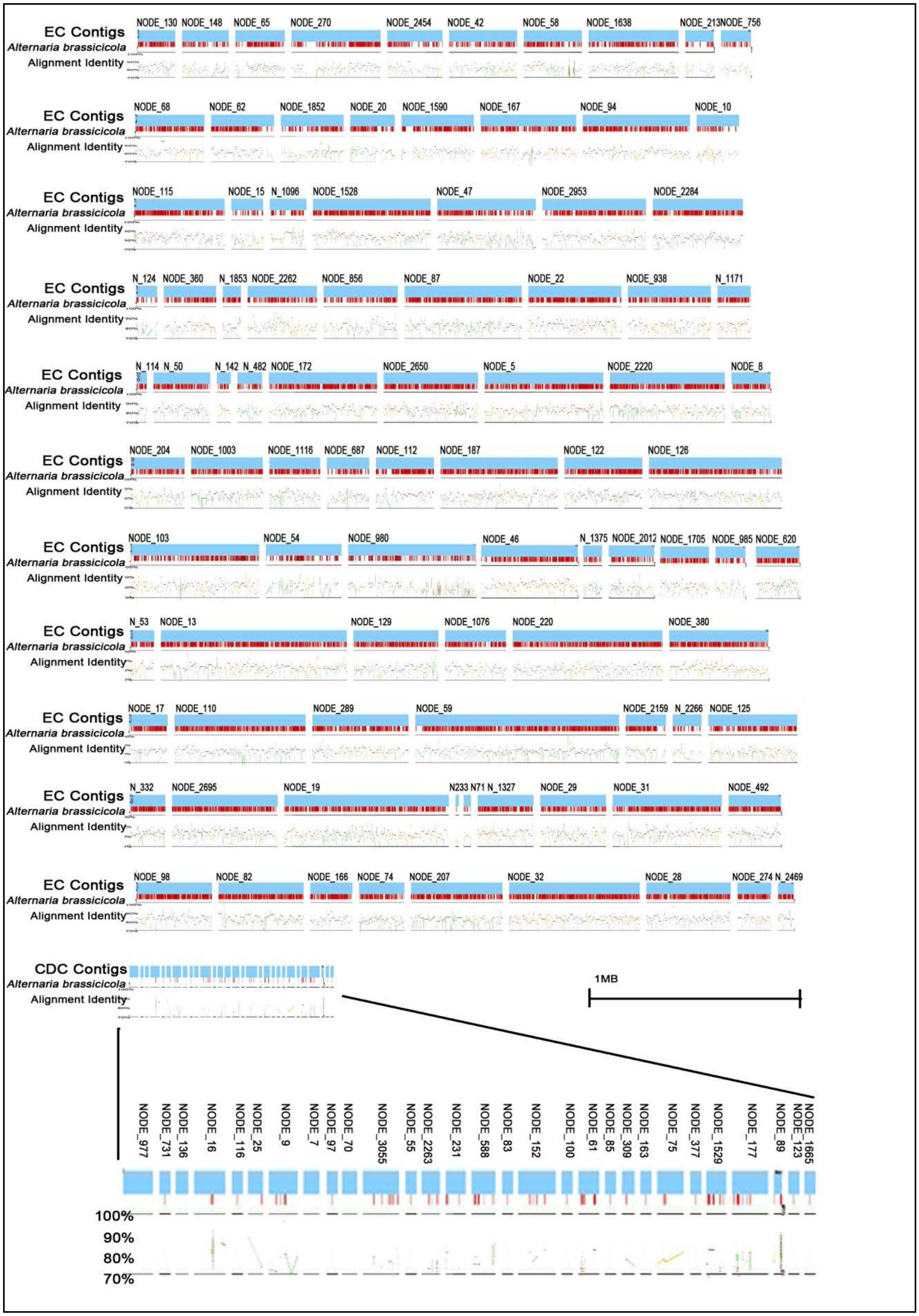

Figure S2. GO term of CDC genes. GO terms were assigned to all CDC genes and level three terms in the categories Biological Process (A), Molecular Function (B), and Cellular Component (C) are showed in the corresponding pie chart.

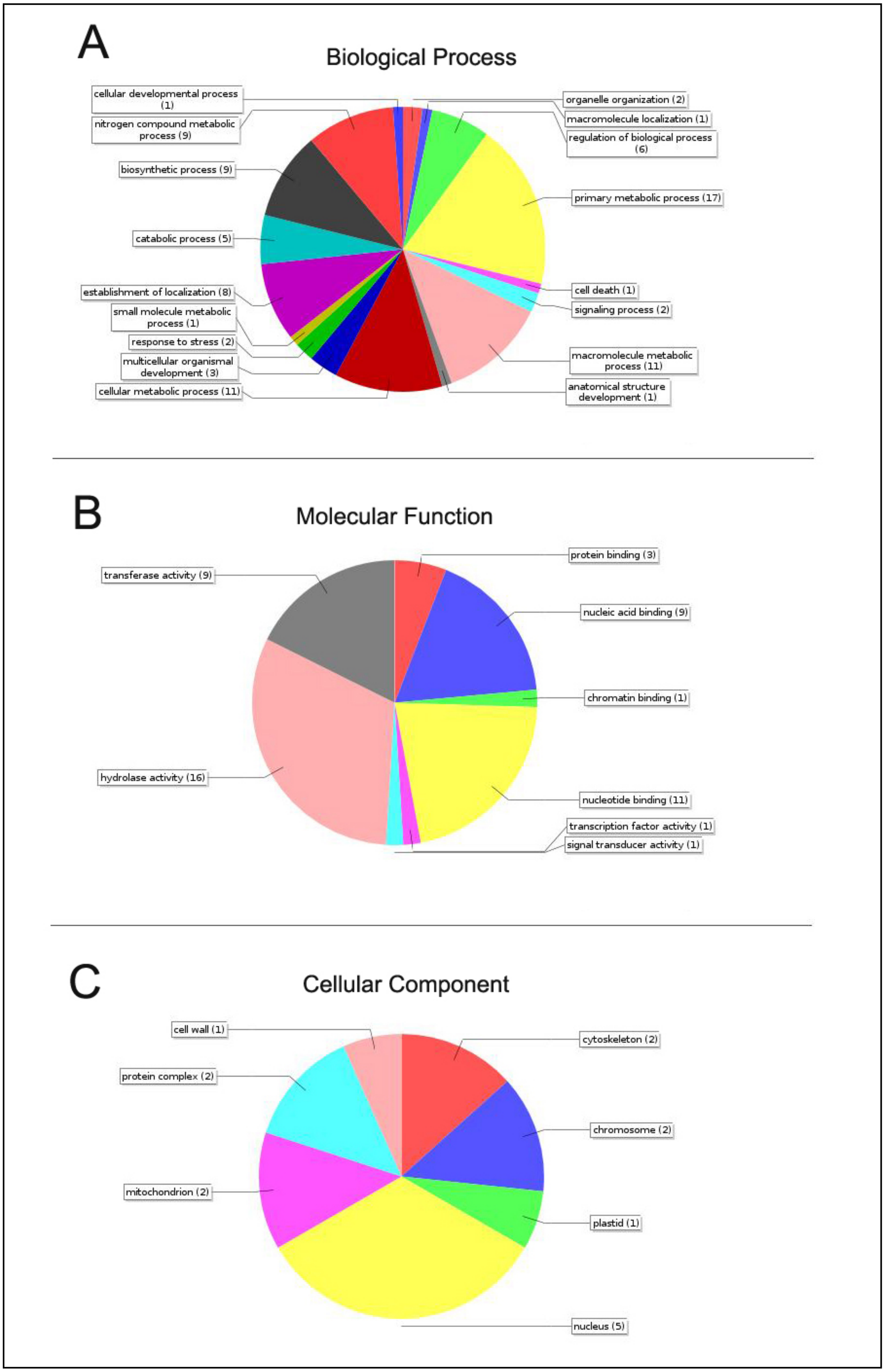

Supplement: Additional file 1 — Supplementary Tables contains: Table S1 to S7. Table S1: Velvet de-novo assembly statistics. Table S2: Repeat region identification. Table S3: Codon usage correlation analysis. Table S4: Differences in codon usage between CDC and EC genes. Table S5: Ka/Ks ratio of CDC protein conversed domains. Table S6: Conserved domains in CDC putative PKS genes. Table S7: Primers used for Southern hybridization. [file 1471-2164-13-171-S1.pdf]
