# Supplementary material for: Genomic characterization of the conditionally dispensable chromosome in Alternaria arborescens provides evidence for horizontal gene transfer
Source: BMC Genomics. 2012 May 6;13:171. doi: 10.1186/1471-2164-13-171 (PMC3443068; doi:10.1186/1471-2164-13-171)
Supplement: Additional file 2 — Supplementary Figures contains: Figure S1 to S2. Figure S1: Global contig alignment between A. arborescens contigs and A. brassicicola contigs. Figure S2: GO term of CDC genes. [file 1471-2164-13-171-S2.pdf]

Table S1. Velvet *de-novo* assembly statistics. Results from assembly attempts using varying parameters. The red data shows the optimized assembly.

K	Ins_length (bp)	Exp_cov	Cut-off	Min_lgth (bp)	N50 (bp)	Max_lgth (bp)	Total size (Mb)	Reads used
**31**	350	42	5	100	84390	485954	33.9	46910423
**41**	350	33	5	100	390559	1219988	33.9	46820556
**51**	350	24	5	100	624394	1544694	34.0	46047345
**57**	350	16	5	100	441130	2135139	34.0	45564058

Table S2. Repeat region identification.

Chromosome	Total length (bp)	Repeat length (bp)	Repeat coverage
**CDC**	906,671	47,990	5.29%
**ECs**	32,675,713	186,054	0.57%

Table S3. Codon usage correlation analysis. Numbers in red show lower correlation scores between CDC and EC contigs compared to correlation within EC groups or within CDC groups, indicating existence of codon usage bias of CDC genes.

	CDC_1	CDC_2	EC_1	EC_2	EC_3	EC_4	EC_5	EC_6	EC_7	EC_8
**CDC_1**	1									
**CDC_2**	0.99498	1								
**EC_1**	0.988367	0.991071	1							
**EC_2**	0.991584	0.994898	0.999034	1						
**EC_3**	0.991876	0.99391	0.99934	0.999358	1					
**EC_4**	0.988976	0.991239	0.999809	0.99915	0.999233	1				
**EC_5**	0.989628	0.991697	0.99981	0.99927	0.999503	0.999778	1			
**EC_6**	0.991425	0.995012	0.998789	0.999853	0.999158	0.998843	0.999091	1		
**EC_7**	0.991919	0.993676	0.999475	0.999374	0.999703	0.999484	0.999566	0.999152	1	
**EC_8**	0.991876	0.99391	0.99934	0.999358	1	0.999233	0.999503	0.999158	0.999703	1

Table S4. Differences in codon usage between CDC and EC genes. Three amino acids showing the most bias.

Codon	Amino Acid	Frequency in CDC	Frequency in EC
**AAA**	K	35.0%	29.6%
**AAG**	K	65.0%	70.4%
**AAT**	N	36.8%	32.3%
**AAC**	N	63.3%	67.7%
**TAT**	Y	37.7%	32.2%
**TAC**	Y	62.4%	67.8%

Table S5. Ka/Ks ratio of CDC protein conversed domains. The last column shows the enrichment of Ka/Ks domains as compared to the whole protein.

domain_ID	Ka	Ks	domain Ka/Ks	protein_Ka/Ks	domain/protein-ratio
CDC_151_HAD_like	67.10	28.90	0.74	0.06	12.33
CDC_151_HMA	51.70	17.30	0.13	0.06	2.10
CDC_84_GAL4	22.00	2.00	0.21	0.11	1.91
CDC_151_E1-E2_ATPase	103.80	49.20	0.10	0.06	1.72
CDC_144_Pat17_PNPLA8_PNPLA9_like	113.50	48.50	0.41	0.30	1.36
CDC_178_AbfB	327.90	107.10	0.12	0.10	1.17
CDC_68_p450	442.30	172.70	0.14	0.14	1.01
CDC_161_RTA1	184.00	77.00	0.12	0.13	0.99
CDC_118_MOZ_SAS	441.40	149.60	0.03	0.03	0.97
CDC_46_TA_like	395.20	153.80	0.10	0.11	0.93
CDC_92_Aa_trans	900.30	293.70	0.07	0.08	0.92
CDC_28_SDR_c	533.30	216.70	0.03	0.03	0.90
CDC_66_P21-Arc	438.40	128.60	0.03	0.04	0.84
CDC_183_Syja_N	916.00	146.00	0.02	0.02	0.84
CDC_169_Rubis-subs-bind	106.90	34.10	0.09	0.12	0.74
CDC_161_Peptidases_S8_S53	256.10	106.90	0.09	0.13	0.71
CDC_102_CAP59_mtransfer	163.00	53.00	0.34	0.51	0.66
CDC_188_Peptidases_S53	957.50	212.50	0.03	0.05	0.64
CDC_178_ArabFuran-catal	629.00	238.00	0.06	0.10	0.60
CDC_67_Rep_fac_C	196.40	70.60	0.02	0.03	0.58
CDC_184_Rab5_related	240.70	56.30	0.01	0.03	0.52
CDC_146_APH	72.30	29.70	0.20	0.45	0.45
CDC_107_PLN02844	469.90	133.10	0.06	0.14	0.44
CDC_161_PA_PoS1_like	245.50	114.50	0.05	0.13	0.42
CDC_161_Peptidases_S8_5	405.40	176.60	0.05	0.13	0.38
CDC_161_DUF1034	237.50	95.50	0.05	0.13	0.37
CDC_51_ACC_central	210.10	68.90	0.01	0.02	0.34
CDC_67_AAA	336.40	116.60	0.01	0.03	0.17
CDC_189_Na_Ca_ex	277.80	88.20	0.00	0.02	0.17

Table S6. Conserved domains in CDC putative PKS genes.

Query	From	To	Bitscore	Accession	Short name
**CDC_4**	2	295	212.038	cl02872	DHQ_Fe-ADH superfamily
**CDC_6**	41	299	123.824	cl10444	Ras_like_GTPase superfamily
**CDC_23**	23	257	189.418	cd05233	SDR_c
**CDC_23**	23	257	189.418	cl09931	NADB_Rossmann superfamily
**CDC_23**	3	259	208.475	PRK05653	fabG
**CDC_28**	50	299	211.374	cd05233	SDR_c
**CDC_28**	50	299	211.374	cl09931	NADB_Rossmann superfamily
**CDC_28**	43	303	220.031	PRK05653	fabG
**CDC_36**	154	381	314.006	cd03249	ABC_MTABC3_MDL1_MDL2
**CDC_36**	154	381	314.006	cl09099	P-loop NTPase superfamily
**CDC_36**	695	934	313.621	cd03249	ABC_MTABC3_MDL1_MDL2
**CDC_36**	695	934	313.621	cl09099	P-loop NTPase superfamily
**CDC_36**	695	923	142.167	COG1126	GlnQ
**CDC_36**	154	370	141.397	COG1126	GlnQ
**CDC_37**	67	496	169.38	cl12078	p450 superfamily
**CDC_41**	457	662	196.457	cl09099	P-loop NTPase superfamily
**CDC_41**	150	688	161.391	COG1132	MdlB
**CDC_42**	694	1102	421.667	cd06206	bifunctional_CYPOR
**CDC_42**	694	1102	421.667	cl06868	FNR_like superfamily
**CDC_42**	26	439	227.546	cl12078	p450 superfamily
**CDC_42**	466	1102	260.325	COG0369	CysJ
**CDC_49**	180	552	285.031	cl00285	Aconitase superfamily
**CDC_80**	1022	1205	116.368	cl00160	LbetaH superfamily
**CDC_141**	33	454	533.223	cd00833	PKS
**CDC_141**	33	454	533.223	cl09938	cond_enzymes superfamily
**CDC_141**	1237	1608	202.13	cd08955	KR_2_FAS_SDR_x
**CDC_141**	1237	1608	202.13	cl09931	NADB_Rossmann superfamily
**CDC_141**	564	879	270.822	cl08282	Acyl_transf_1 superfamily
**CDC_141**	34	457	623.228	smart00825	PKS_KS
**CDC_144**	271	596	127.448	cd07199	Pat17_PNPLA8_PNPLA9_like
**CDC_144**	271	596	127.448	cl11396	Patatin_and_cPLA2 superfamily
**CDC_147**	172	365	121.98	cl14604	Methyltransf_2 superfamily
**CDC_165**	30	447	519.356	cd00833	PKS
**CDC_165**	30	447	519.356	cl09938	cond_enzymes superfamily
**CDC_165**	1909	2144	276.372	cd05195	enoyl_red
**CDC_165**	1909	2144	276.372	cl14614	MDR superfamily
**CDC_165**	2158	2381	171.314	cd08955	KR_2_FAS_SDR_x
**CDC_165**	2158	2381	171.314	cl09931	NADB_Rossmann superfamily
**CDC_165**	640	940	283.123	cl08282	Acyl_transf_1 superfamily
**CDC_165**	1005	1179	110.38	cl11739	PKS_DH superfamily
**CDC_165**	32	449	595.108	smart00825	PKS_KS
**CDC_165**	1913	2144	293.921	smart00829	PKS_ER
**CDC_183**	57	410	355.778	cl11995	Syja_N superfamily

Table S7. Primers used for Southern hybridization.

Probe	Primer_F	Primer_R
**ALT1**	TGCAGTCGAGCTGTCACTTT	GCGATCAGAGATGACGAACA
**CDC_92**	CGTCCGTTATCCTGGTCACT	GAATCGCAGATGCAATGATG
**CDC_102**	CCTCCGCAGCTCTACGATAC	CCTATGCCGTTCCAACAACT
**CDC_147**	ATGATTCGGCAAATCTCTGG	CTTGAGGTAGGCAGGCAAAG
**EC_97_90_g721**	GCCTATCTGCACCGCTCTAC	GATGGCGACTGCTAGACCTC
